# Supplementary material for: The Jacalin-Related Lectin HvHorcH Is Involved in the Physiological Response of Barley Roots to Salt Stress
Source: Int J Mol Sci. 2021 Sep 23;22(19):10248. doi: 10.3390/ijms221910248 (PMC8549704; doi:10.3390/ijms221910248)
Supplement: Supplementary file 1 [file ijms-22-10248-s001.zip › ijms-1374793-supplementary.pdf]

```
                                    ----------------------
                                    ----------------------
HvHorcH                   L   V   P    HA H   R E    DA    K   E HA
horcolin                  P   N   E    PK I   S T    AI    A   V TD
                ******** *** **. ***:  * *:. : :*..  :.:* *** .  .
                -------------------------------
HvHorcH         D  V   -      N       D          Y     V        F
horcolin        V  S   K      T       A          V     -
                 ** .*: **...*.:.*::*: *:******** *::*: ..******:*
                ----------------------------
HvHorcH             Q IEC I    G      T            D        L
horcolin            V KIY -    T      L            A        T
                *.:* *   * :*:* .*:*** .**:*:*****.* ::****:*: *
```

Figure S1. Alignment of the amino acid sequences of horcolin (*HORVU1Hr1G000160*) and HvHorcH (*HORVU7Hr1G059330*) using T-Coffee program [1]. Residues matching the consensus sequence exactly are shaded in black and marked with "*", conserved substitutions are shaded in grey (":") and semi-conserved substitutions are shaded in light grey ("."). The dashed line on top of the alignment indicates the conserved jacalin domain.

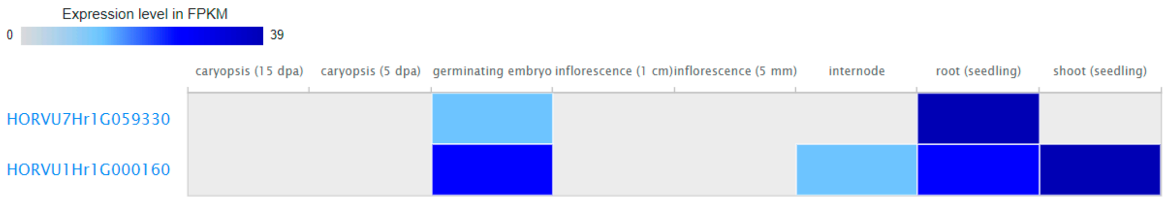

Figure S2. Expression profiles of JRL31 members in barley tissues. The coding sequences of the yet unannotated barley JRL31 *HORVU7Hr1G059330* (named *HvHorcH*) and of the related mannose-specific JRL *HORVU1Hr1G000160* (*horcolin* from barley coleoptiles, [2]) were compared regarding their development and organ specific expression. Analysis was performed using the Expression Atlas (https://www.ebi.ac.uk/gxa/home, accessed on 24th August 2021). FPMK, fragments per kilobase million.

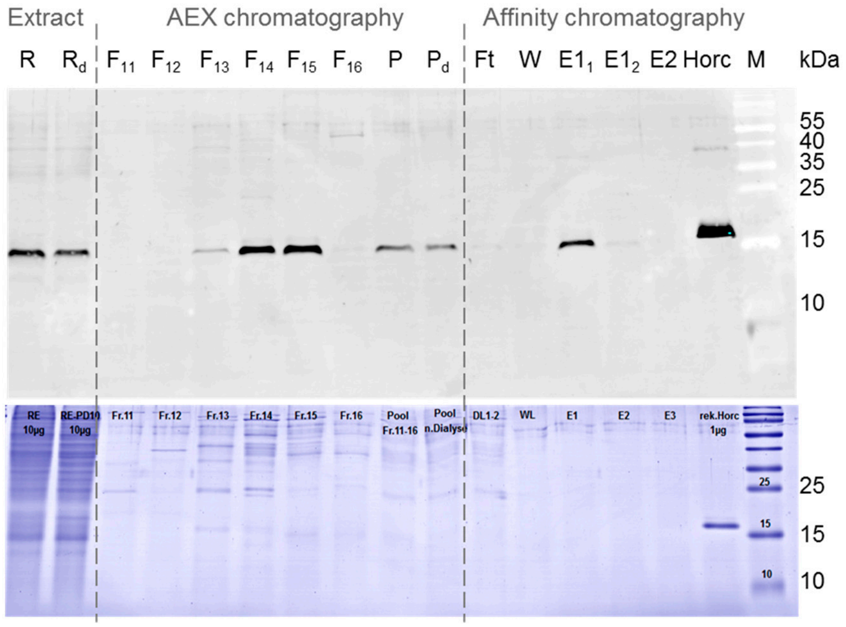

Figure S3. Enrichment of HvHorcH by anion exchange (AEX) and mannose affinity chromatography. Fractions of the various isolation steps were subjected to protein gel blot analysis and abundance of HvHorcH immunologically detected with a polyclonal anti-HvHorcH antibody. A protein band corresponding to HvHorcH was detected at a molecular weight of about 15 kDa in the native root extract (R) and after desalting and buffer exchange (R$_d$). Selective enrichment was observed in fractions from AEX (F$_{13}$ to F$_{16}$) and mannose affinity chromatography (E1$_1$). As a protein control recombinant expressed HvHorcH with a poly-histidine affinity tag was loaded (Horc) producing a signal in the range of 15 to 18 kDa. Loading for protein gel blot analysis (upper blot) and SDS-PAGE (lower gel) was as follows: R—native root extract, 2.5 µg; R$_d$—native root extract after desalting and buffer exchange, 2.5 µg; F$_{11}$ to F$_{16}$—fractions from AEX chromatography, 10 µL; P—pool of AEX F$_{11}$ to F$_{16}$, 10 µL; P$_d$—pool of F$_{11}$ to F$_{16}$ after dialysis, 10 µL; Ft—flow through, 10 µL; W—wash fraction, 10 µL; E1$_1$—first elution with elution buffer 1, 5 µL; E1$_2$—second elution with elution buffer 1, 5 µL; E2—first elution with elution buffer 2, 10 µL; Horc—recombinant HvHorcH, 10 ng; M—marker, PageRuler™ Prestained Protein Ladder, 10 to 180 kDa (Thermo Scientific), 4 µL.

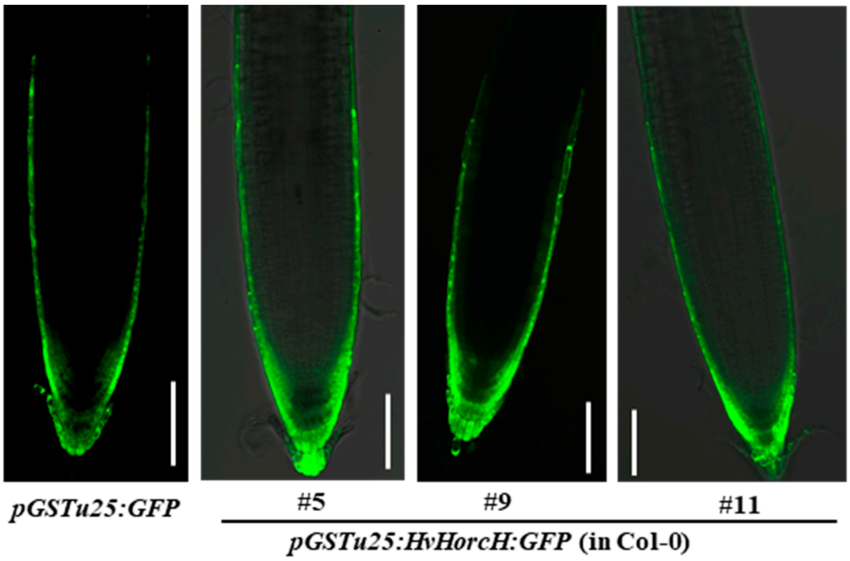

Figure S4. Generation of transgenic Arabidopsis plants expressing HvHorcH. Root tip-specific expression of *HvHorcH:GFP* fusion protein of numerous transgenic lines was verified by confocal microscopy. Representative images of primary root tips of five-day-old seedlings were shown. Scale bars: 100 µm.

Table S1. Protocol for the preparation of barley root tips for immunolocalization of HvHorcH using microwave-assisted fixation, dehydration and embedding in LR White acrylic resin.

<table>
<tr><th colspan="5">Combined conventional & microwave assisted tissue preparation in a
PELCO Bio Wave®Pro+ (Ted Pella, Inc., Redding, USA)</th></tr>
<tr><th>Process</th><th>Reagent</th><th>Power [W]</th><th>Time [sec]</th><th>Vacuum [mm Hg]</th></tr>
<tr><td>1. Primary fixation</td><td>0.5% (v/v) glutaraldehyde
and 2.0% (v/v) paraformaldehyde
in 0.05 M cacodylate buffer (pH 7.3)</td><td>0
150
0
150</td><td>60
60
60
60</td><td>0
0
0
0</td></tr>
<tr><td>2. Wash</td><td>1 × 0.05 M cacodylate buffer (pH 7.3)
and 2x aqua dest.</td><td>150</td><td>45</td><td>0</td></tr>
<tr><td rowspan="2">3. Dehydration</td><td>acetone:30%, 40%, 50%, 60%,
70%, 80%, 90%, 2x 100%</td><td>150</td><td>45</td><td>0</td></tr>
<tr><td colspan="4">after each step samples were kept for additional 15 min on a shaker</td></tr>
<tr><td>4. Resin Infiltration</td><td>25% LR White resin in acetone
50% LR White resin in acetone
75% LR White resin in acetone
100% LR White resin</td><td colspan="3">2 hrs on shaker at RT
2 hrs on shaker at RT
2 hrs on shaker at RT
over night on shaker at RT</td></tr>
<tr><td>5. Polymerization</td><td colspan="4">48 hrs at 60°C in BEEM capsules in a heating cabinet.</td></tr>
</table>

References

1. Di Tommaso, P.; Moretti, S.; Xenarios, I.; Orobitg, M.; Montanyola, A.; Chang, J.M.; Taly, J.F.; Notredame, C. T-Coffee: A web server for the multiple sequence alignment of protein and RNA sequences using structural information and homology extension. *Nucleic Acids Res.* **2011**, *39*, W13–W17.
2. Grunwald, I.; Heinig, I.; Thole, H.H.; Neumann, D.; Kahmann, U.; Kloppstech, K.; Gau, A.E. Purification and characterisation of a jacalin-related, coleoptile specific lectin from *Hordeum vulgare*. *Planta* **2007**, *226*, 225–234.
